# Pathology

**Published:** 1890-11-01

**Authors:** 


### PATHOLOGY.

Dr. Savill first referred to the case of H. A——, which he had described last week, a man who had carcinoma of the liver and some of the fibro-serous membranes, including the dura mater of the spinal cord, which had produced paraplegia ("compression paralsis"). There was, as mentioned, some softening of the cord in the lower dorsal region. Under the microscope it was seen that the grey matter of this region was chiefly affected, and had a very granular appearance.

He then proceeded to describe the case of M. A. C., a woman, aged 64, who was admitted on October 22nd, and died the next morning. On admission she was comatose, and but little history could be found out, beyond the fact that she had had no serious illness for the last three years. The present history was that she was quite well on the Monday; but on the Tuesday at two p.m., she was found on the floor, foaming at the mouth and unconscious. After admission it was noted that all the limbs were flaccid, and there was no movement of the right side, though the left side moved when stimulated; there was no facial paralysis or incontinence either of urine or of fæces. The specific gravity of the urine was 1,012; there was no albumen and no sugar. The breathing was stertorious; the patient vomited later in the evening, and died the next morning.

Post-mortem: The body was well nourished; there was a slight bruise on the right side of the scalp. The serous membranes were normal. In the heart there was slight thickening of the mitral valve; the wall of the left ventricle was also slightly thickened; the musculature was healthy. In the arteries there was some slight thickening of the tunica intima; but it must be remembered in regard to this point that the intima is usually thicker in old than in young people. In this case there was marked thickening of the media, and also of the adventitia, the outline between these two coats not being at all clear. This condition of the vessels corresponds with what is usually described as capillary arterio-fibrosis. The fibrous material is generally described as of a fibro-hyaline character; but this does not appear to be really the case; for if the sections be left for twenty-four hours in logwood stain one finds nuclei of involuntary muscle fibre, and those so distinct that they cannot well be mistaken for anything else. Thus the increase of thickness of the walls appears not to be due to the formation of a fibro-hyaline substance, but to the enlargement of the involuntary muscle fibres themselves. The lungs, liver, and spleen presented no obvious lesion. The kidneys each weighed three and a-half ounces; the capsules were thickened and adherent; and on section of the gland there was seen to be a disproportion between the cortex and medulla. In a normal kidney the relation is of about one to two, but in this case it was one to four or four and a-half. The substance of the organ was firm, and the arteries stood out prominently. Thus they showed an early stage of contracted granular kidney, or the kidney of Bright's disease proper. Microscopically, there was not a very great increase of fibrous tissue, but well-marked thickening of the walls of the vessels. The brain revealed cerebral hæmorrhage. Both ventricles contained blood, which apparently had started on the left side and spread to the right, and had come forward from behind the corpus striatum and optic thalamus.

Speaking of cerebral hæmorrhage, the commonest position for this to occur is a spot just external to the central ganglia, that is to say, in the external capsule. This appears to be the case in about 75 per cent. of the cases, and it occurs from the lenticular striate branch of the middle cerebral artery. In 10 per cent. of the cases it occurs in the pons varolii, and in 10 per cent. in the meninges. At first a clot is produced, which undergoes absorption and becomes organised and leaves what is known as an apoplectic cicatrix, and this may finally undergo cystic degeneration. The causes of cerebral hæmorrhage are not well known pathologically, but they must be, either from an increase in the blood pressure or from a weakening of the vessel at the spot. There is no doubt that atheroma may give rise to cerebral hæmorrhage. Of the seventy-seven cases of central hæmorrhage collected by Charcot and Bouchard, only a quarter showed absence of distinct atheroma of the vessels; but those observers found in every case miliary aneurisms of the terminal branches of the cerebral arteries; and this condition of arteries giving rise to these aneurisms they called sclerous peri-arteritis. It would appear from the description given by the French writers that this is what we know by the term arterio-sclerosis. In these cases there is also frequently some hypertrophy of the left cardiac ventricle, and this condition, together with a rigid vessel, would transmit a very considerable shock with each beat of the heart to the extreme periphery of the vessels, and hence the possibility of aneurism at these spots. So also cerebral hæmorrhage is related to the gouty or granular kidney. About three-fourths of the cases present some such change. This condition of the kidney is not due to increased tension, but to the general arterial changes, and it is these cases in which, with the gouty kidney, the arterial changes are also present. Cerebral hæmorrhage is a disease of age, and is more frequent in the male than in the female, in the proportion of four to five. The old notion that it was essentially a disease of plethoric people with short thick necks and high shoulders is not borne out by recent investigation. It may be produced by a violent effort, and is frequently of an hereditary nature.

The next case described was that of a woman, A. S., aged 68, who was admitted on October 17th, and died on October 23rd. There was nothing special in the family history; she had been ill some four months, and the illness started from a burn. On admission there were marked signs of consolidation at the apex of the left lung. In this region cavernous breathing was heard. The temperature was up and down; once it was 102 at night, and usually normal in the morning. Post-mortem, there was extensive consolidation of the whole of the upper lobe of the left lung, chiefly of a catarrhal pneumonic type; and the centre of this consolidation was the seat of tubercular deposit; the lower lobe also had scattered tubercles of a miliary type. The opposite lung showed early grey tubercles generally scattered throughout. The chief points of interest in the case were the age of the patient for acute pulmonary tuberculosis. It also showed how it is not always possible to diagnose a cavity from solid lung from the auscultatory signs alone; and, again, that the temperature of tuberculosis does not represent the extent, but the activity of the disease. The kidneys had slight thickening of their capsules. The heart was normal. The brain presented an excess of serum on the surface, with slight atrophy of the underlying convolutions; this condition used to be known as serous apoplexy. There was a calcareous deposit in the falx cerebri. The third case was that of a child aged fifteen months. A very debilitated boy, admitted with measles, which were followed by inflammation of the gastro-intestinal tract and aphthous stomatitis. He died of inanition. There was a small calculus found in one of the kidneys, and the mucous membrane of the stomach and small intestine was considerably congested.

